# T-bet^+^ lymphocytes infiltration as an independent better prognostic indicator for triple-negative breast cancer

**DOI:** 10.1007/s10549-019-05256-2

**Published:** 2019-05-08

**Authors:** Hitomi Mori, Makoto Kubo, Masaya Kai, Mai Yamada, Kanako Kurata, Hitomi Kawaji, Kazuhisa Kaneshiro, Tomofumi Osako, Reiki Nishimura, Nobuyuki Arima, Masayuki Okido, Junji Kishimoto, Yoshinao Oda, Masafumi Nakamura

**Affiliations:** 1 Department of Surgery and Oncology, Graduate School of Medical Sciences, Kyushu University, 3-1-1 Maidashi Higashi-ku, Fukuoka, 812-8582 Japan; 2 Breast Center, Kumamoto Shinto General Hospital, Kumamoto, 3-2-65 Ōe Chuo-ku, Kumamoto, 862-8655 Japan; 3 Department of Pathology, Kumamoto Shinto General Hospital, 3-2-65 Ōe Chuo-ku, Kumamoto, 862-8655 Japan; 4 Breast Center, Hamanomachi Hospital, 3-3-1 Nagahama Chuo-ku, Fukuoka, 810-8539 Japan; 5 Department of Research and Development of Next Generation Medicine, Faculty of Medical Sciences, Kyushu University, 3-1-1 Maidashi Higashi-ku, Fukuoka, 812-8582 Japan; 6 Department of Anatomic Pathology, Graduate School of Medical Sciences, Kyushu University, 3-1-1 Maidashi Higashi-ku, Fukuoka, 812-8582 Japan

**Keywords:** T-bet, CD8^+^ lymphocytes, Triple-negative, Biomarker, Prognosis

## Abstract

**Purpose:**

T-box transcription factor 21 (T-bet), which is the master regulator of effector T-cell activation, is derived by stimulation of T-cell receptors. In this study, we focused on T-bet and examined the function of activated T cells.

**Methods:**

This study included 242 patients with primary triple-negative breast cancer (TNBC) who underwent resection without neoadjuvant chemotherapy between January 2004 and December 2014. The immunohistochemistry scoring for CD8 and T-bet expression on tumor-infiltrating lymphocytes (TILs) was defined as ≥ 30 per 6.25 × 10^−3^ mm^2^.

**Results:**

Of the 242 TNBC cases, CD8 was positively expressed in 127 (52.5%) tumors, and T-bet was positively expressed in 67 (27.7%) tumors. T-bet expression was significantly correlated with CD8 expression (*p *< 0.0001). Patients with T-bet^+^ tumors had longer overall survival (OS) compared with patients with T-bet^−^ tumors (*p* = 0.047). The combination of CD8^+^ and T-bet^+^ was associated with a better recurrence-free survival (RFS) and OS compared to CD8^+^/T-bet^−^ tumors (*p *= 0.037 and *p *= 0.024, respectively). Adjuvant chemotherapy provided significantly greater benefit to patients with T-bet^+^ tumors (*p* = 0.031 for RFS, *p* = 0.0003 for OS). Multivariate analysis revealed that T-bet expression on TILs was an independent and positive prognostic indicator (HR = 0.36, 95% confidence interval (CI) 0.12–0.94, *p* = 0.037 for RFS, HR = 0.30, 95% CI 0.07–0.95, *p* = 0.039 for OS).

**Conclusions:**

OS was significantly improved for patients with high T-bet-expressing TILs in TNBC. Thus, T-bet may be a predictive indicator for survival and various immunotherapy strategies in TNBC.

**Electronic supplementary material:**

The online version of this article (10.1007/s10549-019-05256-2) contains supplementary material, which is available to authorized users.

## Introduction

Recent studies have shown that the tumor immune system plays an important role in solid tumors microenvironment (TME). In the TME, an immune response requires tumor-associated antigen (neoantigen) signatures presented through accumulation of gene mutations [1]. That is, tumor-infiltrating lymphocytes (TILs) frequently recognize neoantigens from a tumor, and then some of TILs become neoantigen-reactive T cells for cytotoxicity [2, 3]. Therefore, a high tumor-mutational burden (TMB) derives enhanced clinical benefit from immune check point inhibitors [4]. Meanwhile, triple-negative breast cancer (TNBC) lacking the expression of estrogen and progesterone receptors and ERBB2 is a heterogeneous tumor that encompasses several molecular subtypes of breast cancer. Because this specific subtype of TNBC includes high levels of somatic mutations [5], it is expected to benefit from a variety of immunotherapies. Many analyses of treatment for immune checkpoint blockade have made it clear that TILs play an important role in treating cancers in both adjuvant and neoadjuvant settings [6–8]. We previously reported that programmed cell death ligand-1 (PD-L1) expression on tumor cells was related to high TIL levels, and the combination of high TIL levels and positive PD-L1 was associated with a better prognosis in TNBC [9]. However, the molecular mechanism remains still unclear.

TILs are frequently observed but the composition of cells involved in innate and adaptive immunity varies between tumor types or organ sites [10]. Cumulative data from murine and human studies have associated most leukocyte subsets with a predominant contribution to either pro- or antitumor activities [11]. For instance, effector CD8^+^ T cells and CD4^+^ T cells affect immunity, while regulatory T cells influence tolerance [12]. It was reported that the ratio of CD8^+^ cytotoxic T lymphocytes to FOXP3^+^ regulatory T cells (Tregs) in tissue surrounding tumors was an independent prognostic factor for breast cancer and was associated with the prognosis of the molecular subtypes of tumors [13].

T-bet (encoded by *TBX21*) is an immune cell-specific member of the T-box family of transcription factors. T-bet is expressed in multiple immune cells including dendritic cells, natural killer cells, CD4^+^ and CD8^+^ effector cells, B cells and a subset of Tregs, and plays a pivotal role in infectious, inflammatory and autoimmune conditions, such as Crohn’s disease, type 1 diabetes, allergic asthma, rheumatoid arthritis, multiple sclerosis and so on [14], as well as in TME. T-bet is upregulated by stimulation of T-cell receptors and IL-12, and then regulates effector T-cell activation. Activated T cells function as antitumor lymphocytes by enhancing the production of cytokines such as INFγ [15]. Previous studies showed that high numbers of T-bet^+^ intratumoral lymphoid cells have been found to correlate with an improved outcome in gastric cancer [16], colorectal cancer [17] and in high-grade cervical intraepithelial neoplasia [18]. Furthermore, in a cohort of woman with familial breast cancer, T-bet^+^ lymphocytes were associated with the basal molecular subtype as well as with morphological features characteristic of such tumors, including high tumor grade, p53 expression, ER-negativity, CK5-positivity and EGFR-positivity, and also were correlated with a good prognosis [19]. In addition, T-bet^+^ TILs were associated with a favorable outcome in all breast cancers [20]. However, there are few reports regarding the relationship between T-bet expression and prognosis, or between effector CD8^+^ T cells and T-bet in TNBC.

In the present study, we retrospectively analyzed CD8 and T-bet expression on lymphocytes in 242 TNBC cases. We also explored the correlation between immune system features, including T-bet positivity, clinicopathologic characteristics, response to chemotherapies and clinical outcome.

## Methods

### Patients and treatments

This study included 242 patients with primary TNBC who underwent resection without neoadjuvant chemotherapy at Kyushu University Hospital (Fukuoka, Japan), Hamanomachi Hospital (Fukuoka, Japan) or Kumamoto City Hospital (Kumamoto, Japan) between January 2004 and December 2014. Approximately, 20% of TNBC patients received neoadjuvant chemotherapy and were excluded from this study. The patients were treated according to the National Comprehensive Cancer Network Guidelines for treatment of breast cancer [21], the recommendations of the St. Gallen International Breast Cancer Conference [22–25] and the Clinical Practice Guidelines for Breast Cancer by the Japanese Breast Cancer Society [26]. The adjuvant treatments for the patients are shown in Supplementary Table S1. The study conformed to the principles of the Declaration of Helsinki and was approved by the Institutional Review Board (IRB) of Kyushu University Hospital (No. 30-231). Prior to their operations, participants comprehensively provided their written consent stating that the tissue samples from resected specimen may be used for various researches. Once the IRB approved this study, all details were made available on the Kyushu University Hospital website instead of renewing informed consent. All patients have the option to confirm ongoing studies and may choose to opt out of consent at any time. The IRB approved this consent procedure.

### Immunohistochemistry (IHC) staining

Tumor subtypes were identified using IHC staining on surgically resected tissue. All resected specimens used for IHC were fixed (fixation was begun within 1 h) in 10% neutral buffered formalin for 6–72 h. ER-positive or PR-positive tissues were defined as ≥ 1% of tumor cells staining positive for ER or PR. Cancer specimens were defined as HER2 positive when HER2 IHC staining was scored as 3+ according to the standard criteria [27, 28], or when *HER2* gene amplification was detected using fluorescence spectroscopy with in situ hybridization.

Primary anti-CD8 antibody (monoclonal mouse, C8/144B; Nichirei Bioscience Inc., Tokyo, Japan) was used according to the protocol. Briefly, slides were deparaffinized and immersed in unmasking solution (pH 6.0) at a sub-boiling temperature (95–98 °C) for heat-induced antigen unmasking. The primary antibody was used at no dilution and was incubated for 60 min, and the secondary anti-mouse antibody was incubated for 40 min at room temperature. Slides were counterstained with hematoxylin. Primary anti-T-bet antibody (monoclonal rabbit, D6N8B; Cell Signaling Technology, Beverly, MA) was used at a 1:1600 dilution and incubated overnight at 4 °C, and the secondary anti-rabbit antibody was incubated for 40 min at room temperature. CD8^+^ and T-bet^+^ staining was evaluated according to previous reports [20]. Briefly, tumor-infiltrating CD8^+^ T lymphocytes were counted separately according to their intracellular localization, i.e. intraepithelial (intratumoral) or stromal. CD8^+^ TILs were counted under a microscopic field at × 200 magnification (0.00625 mm^2^). Five areas with the most abundant infiltration were selected, and the average count was calculated. The results were interpreted as positive when more than or equal to 30 cells per 0.0625 mm^2^ were identified in intraepithelial (intratumoral) or stromal areas (Supplementary Fig. S1a). T-bet^+^ lymphocytes were evaluated in the same way (Supplementary Fig. S1b). PD-L1 expression on tumor cells was evaluated according to our previous report [9].

### Statistics

Logistic regression was used to compare continuous variables and χ^2^ tests were used to compare categorical variables between T-bet^+^ and T-bet^−^ groups. The survival endpoints evaluated were recurrence-free survival (RFS) and overall survival (OS). RFS was defined as the time from surgery to recurrence, including both local relapse and metastatic disease. OS was defined as the time from surgery until the date of death from any cause. Survival curves were generated using the Kaplan–Meier method and compared with the log-rank test. Interactions between T-bet and other factors were evaluated using nested effects in the Cox proportional hazards model. Variables for the multivariate analysis were selected through the back elimination method. However, variables, which were known as prognostic factors and highly associated with T-bet, were included in the multivariate analysis. Hazard ratios (HR) were calculated using Cox proportional hazards regression. Values of *p* < 0.05 were considered statistically significant. The multiplicity was not adjusted for RFS and OS because this research was developing and exploratory. Statistical analysis was carried out using JMP 11 (SAS Institute Inc., Cary, NC).

## Results

### Clinicopathologic features and CD8 and T-bet expression

We evaluated 242 TNBC tumors with respect to the clinicopathologic data (Table 1) and CD8 and T-bet expression on TILs (Supplementary Fig. S1). Among the 242 TNBC cases, CD8 on TILs was expressed as positive in 127 (52.5%) tumors (Supplementary Table S2) and T-bet on TILs was expressed as positive in 67 (27.7%) tumors (Table 1). When focusing on T-bet expression, T-bet^+^ tumors were smaller than T-bet^−^ tumors (*p* = 0.04), and there was no significant difference in nodal status and pathological stage between T-bet^+^ and T-bet^−^ tumors (Table 1). Nuclear grade and Ki-67 labeling index in T-bet^+^ tumors were higher than in T-bet^−^ tumors (*p *< 0.0001 and *p *< 0.0001, respectively; Table 1). Analysis of the combination of CD8 and T-bet expression revealed T-bet was positive in 55 (22.7%) CD8^+^ tumors and 12 (5.0%) CD8^−^ tumors, and T-bet was negative in 72 (29.7%) CD8^+^ tumors and 103 (42.6%) CD8^−^ tumors. T-bet expression on TILs was significantly correlated with CD8 expression on TILs (Table 1). T-bet expression on TILs was also significantly correlated with PD-L1 expression on tumor cells (Table 1).

**Table 1:** Clinicopathologic characteristics

	T-bet^+^	T-bet^−^	*p* value
	*N* = 67 (27.7%)	*N* = 175 (72.3%)	
Age at diagnosis			
Mean (range)	58.2 (32–86)	60.8 (30–89)	0.16^a^
Tumor size			
T1a/b (≤ 1 cm)	6 (8.9%)	14 (8.0%)	**0.04** ^b^
T1c (> 1 cm, ≤ 2 cm)	42 (62.7%)	79 (45.1%)	
T2 (> 2 cm, ≤ 5 cm)	19 (28.4%)	75 (42.9%)	
T3 (> 5 cm)	0	7 (4.0%)	
Nodal status			
N0	44 (65.7%)	118 (67.4%)	0.62^b^
N1 (1–3)	19 (28.3%)	39 (22.3%)	
N2 (4–9)	2 (3.0%)	11 (6.3%)	
N3 (≥ 10)	2 (3.0%)	6 (3.4%)	
Unknown		1 (0.6%)	
Pathological stage			
I	31 (46.3%)	71 (40.6%)	0.33^b^
II	33 (49.2%)	86 (49.1%)	
III	3 (4.5%)	18 (10.3%)	
Nuclear grade			
1 + 2	5 (7.5%)	65 (37.2%)	**< 0.0001** ^b^
3	58 (86.5%)	107 (61.1%)	
Unknown	4 (6.0%)	3 (1.7%)	
Ki-67 (%)			
≤ 30	3 (5.8%)	45 (29.0%)	**< 0.0001** ^b^
> 30	57 (80.6%)	104 (57.9%)	
Unknown	7 (13.6%)	26 (13.1%)	
CD8			
Negative	12 (17.9%)	103 (58.9%)	**< 0.0001** ^b^
Positive	55 (82.1%)	72 (41.1%)	
PD-L1 on tumor cells			**< 0.0001** ^b^
Negative	22 (32.8%)	121 (69.1%)	
Positive	45 (67.2%)	54 (30.9%)	
Surgical treatment			
Breast-conserving surgery	50 (74.6)	94 (53.7)	**0.003** ^b^
Mastectomy	17 (25.4)	81 (46.3)	
Adjuvant treatment			
Chemotherapy	50 (74.6%)	117 (66.8%)	0.27^b^
No treatment	17 (25.4%)	57 (32.6%)	
Unknown	0	1 (0.6%)	

Bold value represents that *P* value was significant

^a^Logistic regression

^b^Pearson’s *χ*^2^ test

### Patient survival

**Fig. 1:**
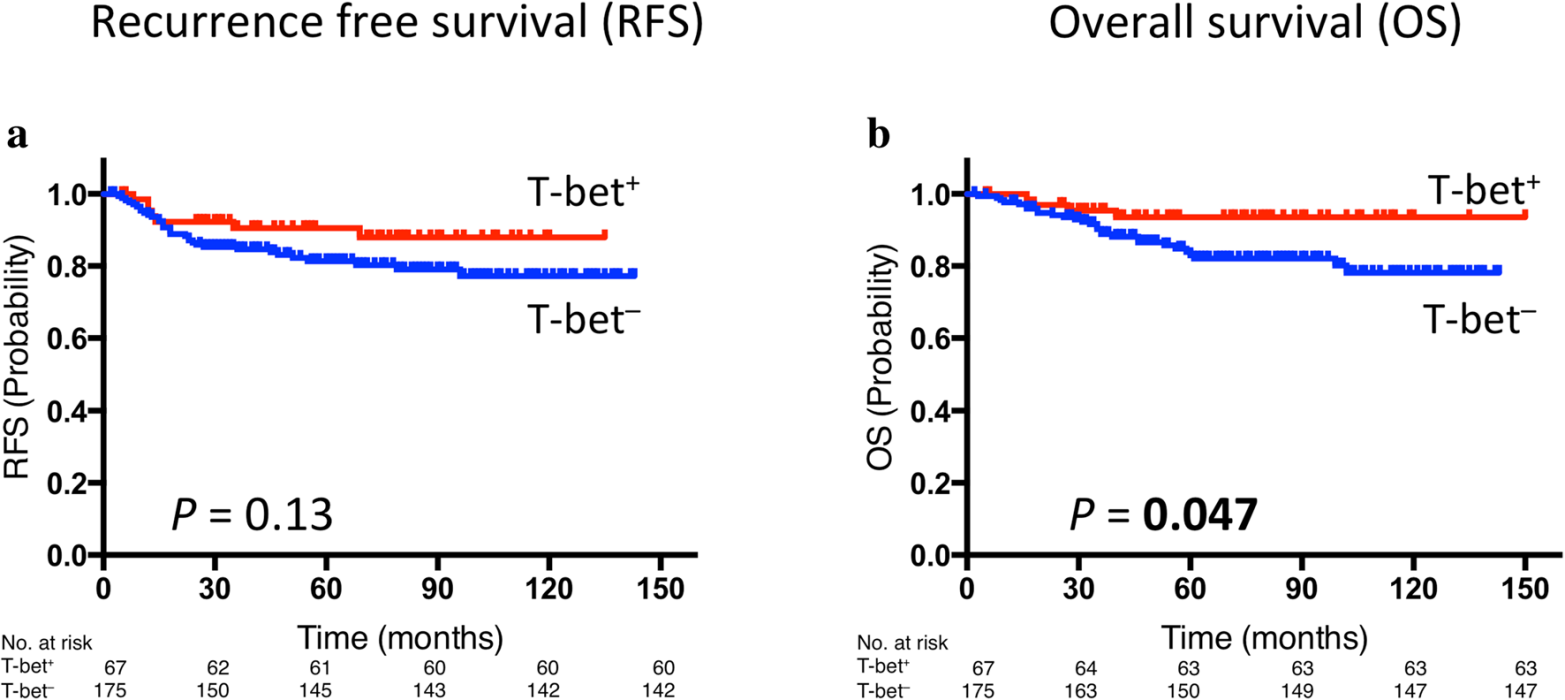
Prognostic value of T-bet expression: Kaplan–Meier curves showing estimated RFS (**a**) and OS (**b**) for T-bet expression. *p* values are for comparison of two groups

The median follow-up in this cohort was 67 months (range 2–150 months). There was no significant difference in RFS and OS between patients with CD8^+^ tumors and those with CD8^−^ tumors (Supplementary Fig. S2). Although there was no significant difference in RFS between patients with T-bet^+^ tumors and those with T-bet^−^ tumors (Fig. 1a), patients with T-bet^+^ tumors had significantly better OS than those with T-bet^−^ tumors (*p* = 0.047, Fig. 1b).

**Fig. 2:**
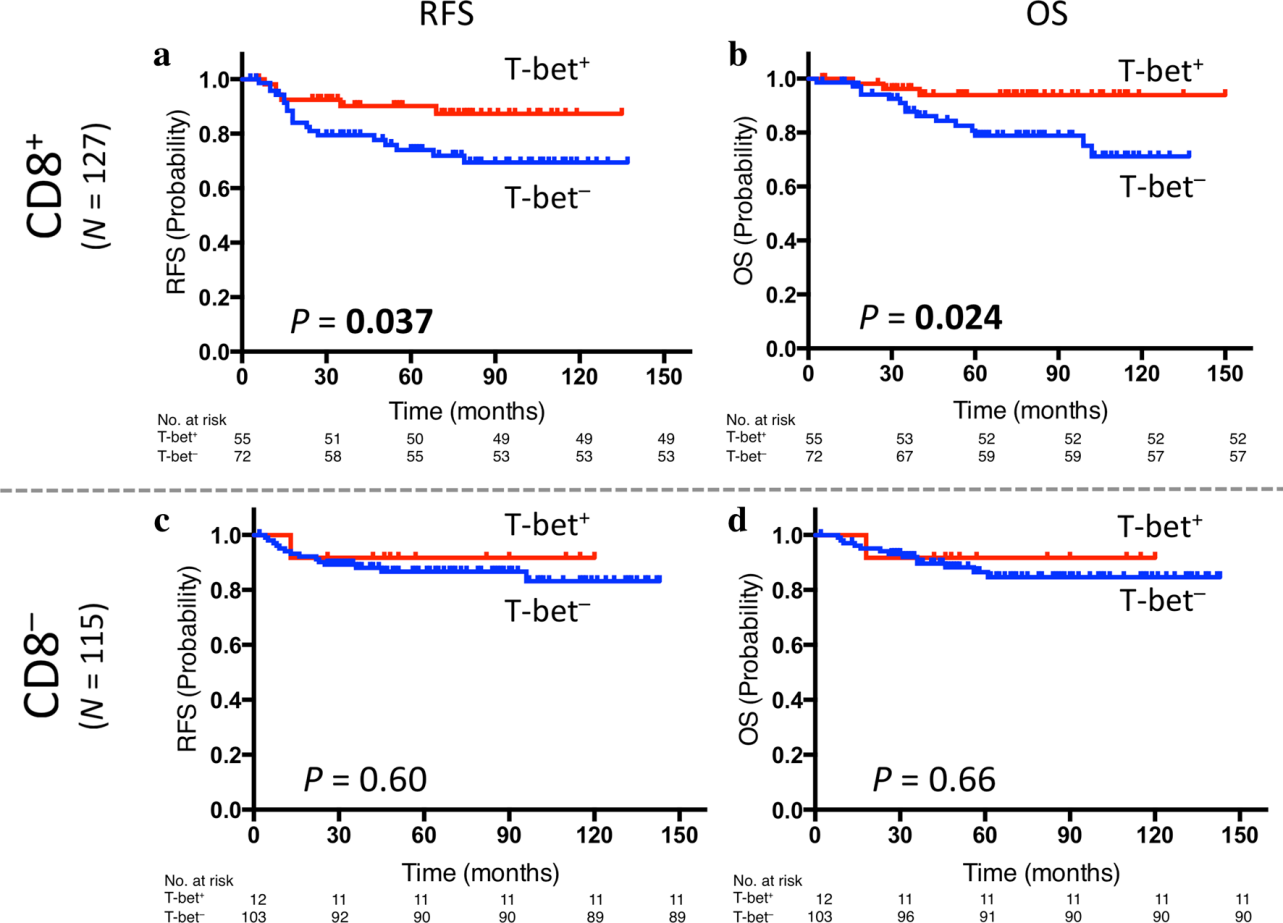
Prognostic value of the combination of CD8 and T-bet expression: Kaplan–Meier curves showing estimated RFS (**a**) and OS (**b**) for T-bet expression in CD8-positive tumors as well as RFS (**c**) and OS (**d**) for T-bet expression in CD8-negative tumors. *p* values are for comparison of two groups

Next, we evaluated the prognosis of patients categorized according to the combination of CD8 and T-bet expression. When focusing on CD8^+^ tumors, patients with CD8^+^/T-bet^+^ tumors had significantly better RFS and OS than those with CD8^+^/T-bet^−^ tumors (*p *= 0.037 and *p *= 0.024, respectively, Fig. 2a, b). Meanwhile, in the case of CD8^−^ tumors, there was no significant difference between CD8^−^/T-bet^+^ tumors and CD8^−^/T-bet^−^ tumors (Fig. 2c, d). The adjuvant treatment background of these four subgroups did not significantly differ (Supplementary Table S1). Furthermore, the results of nested effects in the Cox proportional hazards model showed that the effect of T-bet occur only within CD8^+^ tumors when we evaluated interactions between T-bet and CD8 (Supplementary Table S3a).

### Adjuvant chemotherapy and clinical outcome

**Fig. 3:**
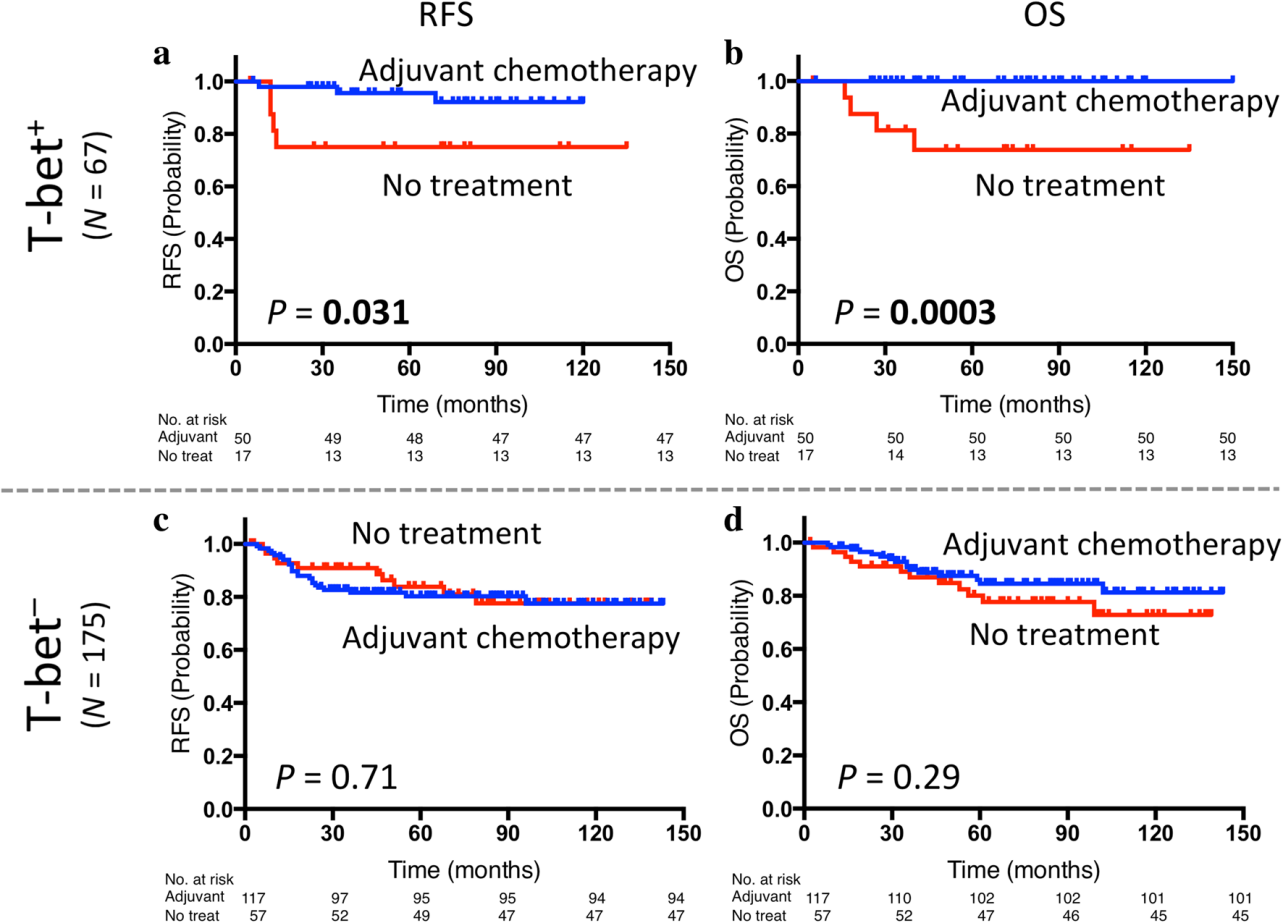
Prognostic value of adjuvant chemotherapy by T-bet: Kaplan–Meier curves showing estimated RFS (**a**) and OS (**b**) for treatment status in T-bet-positive tumors as well as RFS (**c**) and OS (**d**) for treatment status in T-bet-negative tumors. *p* values are for comparison of two groups. *Adjuvant* adjuvant chemotherapy, *No treat* no treatment

Among the 242 TNBC patients, 167 (69.0%) patients received adjuvant chemotherapy, 74 (30.6%) patients received no treatment, and there was no information available for 1 (0.4%) patient (Table 1). Adjuvant chemotherapy provided significantly greater benefit to patients with T-bet^+^ tumors (*p* = 0.031 for RFS, *p* = 0.0003 for OS, Fig. 3a, b). In patients with T-bet^−^ tumors, their prognosis did not significantly differ between the patients who received adjuvant chemotherapy and those who received no treatment (Fig. 3c, d). In addition, the results of nested effects showed that the effect of adjuvant chemotherapy occurs only within T-bet^+^ tumors when we evaluated interactions between T-bet and treatment (Supplementary Table S3b).

### Univariate and multivariate survival analysis

Univariate analysis of the clinicopathologic characteristics revealed that tumor size (> 2 cm) and lymph node involvement were significantly related to poorer RFS and OS, while receiving adjuvant chemotherapy and T-bet^+^ tumors were significantly related to better OS (Table 2a). Multivariate analysis revealed that the tumor size (> 2 cm) was a negative prognostic factor for RFS and that the lymph node involvement was also a negative prognostic factor for both RFS and OS. Adjuvant chemotherapy provided significantly better OS. Meanwhile, T-bet expression proved to be an independent positive prognostic factor for both RFS and OS (HR = 0.36, 95% confidence interval (CI) 0.12–0.94, *p* = 0.037 for RFS, HR = 0.30, 95% CI 0.07–0.95, *p* = 0.039 for OS) (Table 2b).

**Table 2:** Cox proportional hazards model for recurrence-free and overall survival

Variables		Recurrence-free survival			Overall survival		
		RR	95% CI	*p* value	RR	95% CI	*p* value
A univariate analysis							
Age	> 50 versus ≤ 50	1.16	0.58–2.59	0.69	1.45	0.64–3.88	0.40
Tumor size	> 2 cm versus ≤ 2 cm	2.65	1.41–5.14	**0.0023**	2.33	1.16–4.84	**0.018**
Nodal status	Positive versus negative	2.75	1.48–5.20	**0.0015**	2.26	1.12–4.56	**0.023**
Nuclear grade	3 versus 1 and 2	1.03	0.52–2.17	0.94	0.69	0.34–1.45	0.32
Ki-67	> 30% < versus ≤ 30%	1.61	0.57–6.70	0.41	0.75	0.31–2.23	0.58
CD8	Positive versus negative	1.52	0.81–2.96	0.20	1.10	0.55–2.26	0.78
PD-L1 on TC	Positive versus negative	0.84	0.43–1.58	0.60	0.62	0.28–1.27	0.20
Adjuvant treatment	Chemo. versus no treat.	0.80	0.42–1.57	0.51	0.43	0.22–0.87	**0.02**
T-bet	Positive versus negative	0.54	0.22–1.14	0.11	0.36	0.11–0.92	**0.032**
B Multivariate analysis							
Tumor size	> 2 cm versus ≤ 2 cm	2.73	1.29–6.19	**0.0084**	2.18	1.00–5.05	0.05
Nodal status	Positive versus negative	3.17	1.50–6.92	**0.0024**	2.78	1.26–6.24	**0.012**
Nuclear grade	3 versus 1 and 2	0.99	0.42–2.58	0.98	0.58	0.25–1.43	0.24
Ki-67	> 30% < versus ≤ 30%)	1.79	0.62–6.02	0.30	1.90	0.70–5.78	0.22
CD8	Positive versus negative	2.00	0.95–4.33	0.07	1.52	0.69–3.32	0.30
Adjuvant treatment	Chemo. versus no treat.	0.67	0.32–1.43	0.29	0.39	0.18–0.85	**0.018**
T-bet	Positive versus negative	0.36	0.12–0.94	**0.037**	0.30	0.07–0.95	**0.039**

Bold value represents that *P* value was significant

*HR* hazard ratio, *CI* confidence interval, *TC* tumor cells, *Chemo* chemotherapy, *No treat* no treatment

## Discussion

According to the reports by Denkert et al. increased levels of TILs in woman receiving neoadjuvant chemotherapy were associated with improved prognosis in HER2+ or TNBC, but a poorer outcome in ER+/HER2− breast cancer [7]. In addition, in our previous study, we evaluated TILs in TNBC according to international TILs guidelines, and our data showed that patients with TILs-high tumors had significantly better OS than those with TILs-low tumors [6]. This finding was consistent with the previous results, which showed that each 10% increase in TILs strongly predicted better survival [29]. To clarify the biological function of TILs, which is a host factor in TME, we focused on effector CD8^+^ T cells and transcription factor T-bet.

Our results showed that CD8-positivity in TNBC was 52.5% (Supplementary Table S2). In previous studies, CD8-positivity of intratumoral and stromal CD8^+^ TILs in all subtypes of breast cancer ranged from 47.5 to 79.1% [13, 30, 31], and CD8 expression was associated with ER-negative status. CD8^+^ T cells represent a candidate biomarker of the tumor-associated immune response as a major component of the adaptive immune system. Compelling evidence point to clinical relevance for high numbers of T cells at the tumor site, with CD8^+^ T cells as a critical denominator for OS in patients with colorectal cancer [32], and also for other solid tumors. In this TNBC study, CD8 expression by itself was not a predictive factor. Most previous studies regarding CD8^+^ T cells in all subtypes of breast cancer have reported an association with favorable outcomes [30, 31, 33, 34], but others have not [13].

T-bet expression on TILs was a good prognostic factor for node-negative breast cancer including all subtypes [20, 35]. However, T-bet-positivity in TNBC is rarely reported and it is still unclear whether T-bet expression is correlated with breast cancer subtype. We focused on TNBC in this study and found that T-bet-positivity was 27.7%. Small tumor size, high nuclear grade, high Ki-67 and breast-conserving surgery were significantly correlated with T-bet expression. The reason why patients with T-bet^+^ tumors received more breast-conserving surgery compared with those with T-bet^−^ tumors is probably because tumor size was smaller. However, there was no significant difference in nodal status and pathological stage between T-bet^+^ and T-bet^−^ tumors. Mulligan et al. reported that T-bet^+^ tumors were associated with a large tumor size [20], in contrast to our result. However, we are considering the possibility that the tumor immune system cannot function well when tumors are too large. While in small-sized tumors, T-bet expression might be high and effector T cells work well.

We also indicated that OS was significantly longer among patients with high T-bet-expressing TNBC. T-bet is the recognized lineage-defining transcription factor and mediates direct, positive feed-forward regulation of INFγ production for Th1 cells [36]. Recent studies suggested that the development of Th1 adaptive immunity was associated with improved outcome in various cancer types [37, 38]. Therefore, T-bet may become a predictive factor for better prognosis of various cancer types. In addition, T-bet expression on TILs was significantly associated with PD-L1 expression on tumor cells (Table 1). That was because PD-L1-amplified tumors were classified as having high TMB compared with unamplified tumors [39].

When verified at the mRNA level using KM plotter, which is the public data from Gene Expression Omnibus (National Cancer for Biotechnology Information, Bethesda, MD) database, either CD8-high or T-bet-high mRNA expression was significantly correlated with longer RFS among TNBC patients (Supplementary Fig. S3).

In addition, we indicated that T-bet expression was associated with response to chemotherapy, and this is the first report of a relationship between T-bet and response to conventional chemotherapy including anthracycline and taxane in TNBC. The relationship between T-bet and response to chemotherapy has not been investigated in many cancer types, and only one study showed that T-bet expression in intratumoral lymphoid structures after neoadjuvant trastuzumab–taxane in HER2-overexpressing breast cancer predicted better outcome [40]. This study included both patients who had been treated with trastuzumab–taxane and anthracycline-based neoadjuvant chemotherapy, and the presence of T-bet^+^ TILs after chemotherapy conferred significantly better RFS (*p* = 0.011) only in patients treated with trastuzumab–taxane [40]. Although further investigation is needed, T-bet expression on TILs may become a predictive factor in response to chemotherapy or molecular targeted treatment.

This is also the first report of an interaction between T-bet and CD8 expression in breast cancer. T-bet expression was significantly correlated with CD8 expression on TILs. Even if CD8^+^ TILs existed in the TME, patients with T-bet^−^ tumors had a significantly worse prognosis. Prognosis was improved when CD8^+^ effector T cells were present in the TME and T-bet was also expressed on immune cells. The presence of TILs in the TME is necessary to improve patient survival. However, not only the presence of TILs but also whether T cells are functioning or not may be important. Thus, the combination of T-bet and CD8 may be the most robust predictive factor of prognosis in TNBC.

This study had several limitations. First, it included only retrospectively collected cases. Second, the sample size was small. Although we assessed the interactive effect of T-bet and CD8, the causal relationship is not clear. We revealed that T-bet might be a prognostic or predictive factor. Since this study is developing and exploratory, we are planning the next study to make sure that T-bet will be a biomarker; for instance, a translational research using samples from the clinical trial of TNBC that has already been completed, or that will start in the future. Our final goal is to identify biomarkers that are functional indicators of tumor immune activation and also predictive factors in terms of treatment effect or resistance for immune checkpoint inhibitors.

## Conclusions

Our findings suggested that T-bet expression on TILs is significantly correlated with CD8 expression on TILs and associated with better prognosis in patients with TNBC. OS is significantly longer among patients with high T-bet-expressing TNBC. These results may validate the significance of T-bet as an indicator for various immunotherapies in TNBC.

## Electronic supplementary material

Below is the link to the electronic supplementary material.

Supplementary Fig. S1: Immunostaining of CD8 and T-bet on TILs (×400). (TIFF 26,330 kb)

Supplementary Fig. S2: Prognostic value of CD8 expression: Kaplan-Meier curves showing estimated RFS (a) and OS (b) for CD8 expression. *p* values are for comparison of two groups. (TIFF 26,330 kb)

Supplementary Fig. S3: Prognostic value of CD8 and T-bet mRNA expression in KM plotter (kmplot.com). Kaplan-Meier curve showing estimated RSF for CD8 (**a**) and T-bet (**b**) mRNA expression from triple negative breast cancer patients (N = 255) (TIFF 26,330 kb)

Supplementary Table S1: Treatment characteristics for patients with TNBC. (DOCX 85 kb)

Supplementary Table S2: Patient and tumor characteristics based on CD8 expression. (DOCX 104 kb)

Supplementary Table S3: Interaction between T-bet and other factors in a Cox proportional hazards model (DOCX 66 kb)

## Abbreviations

TME: Tumor microenvironment
TILs: Tumor-infiltrating lymphocytes
TMB: Tumor-mutational burden
TNBC: Triple-negative breast cancer
PD-L1: Programmed cell death ligand-1
Tregs: Regulatory T cells
IRB: Institutional review board
IHC: Immunohistochemistry staining
RFS: Recurrence-free survival
OS: Overall survival
HR: Hazard ratio
CI: Confidence interval

## References

[1] Matsushita H, Vesely MD, Koboldt DC (2012). Cancer exome analysis reveals a T-cell-dependent mechanism of cancer immunoediting. Nature.

[2] Robbins PF, Lu YC, El-Gamil M (2013). Mining exomic sequencing data to identify mutated antigens recognized by adoptively transferred tumor-reactive T cells. Nat Med.

[3] Cohen CJ, Gartner JJ, Horovitz-Fried M (2015). Isolation of neoantigen-specific T cells from tumor and peripheral lymphocytes. J Clin Invest.

[4] Bouffet E, Larouche V, Campbell BB (2016). Immune checkpoint inhibition for hypermutant glioblastoma multiforme resulting from germline biallelic mismatch repair deficiency. J Clin Oncol.

[5] The Cancer Genome Atlas Network (2012). Comprehensive molecular portraits of human breast tumours. Nature.

[6] Wang K, Xu J, Zhang T (2016). Tumor-infiltrating lymphocytes in breast cancer predict the response to chemotherapy and survival outcome: a meta-analysis. Oncotarget.

[7] Denkert C, von Minckwitz G, Darb-Esfahani S (2018). Tumour-infiltrating lymphocytes and prognosis in different subtypes of breast cancer: a pooled analysis of 3771 patients treated with neoadjuvant therapy. Lancet Oncol.

[8] Pelekanou V, Carvajal-Hausdorf DE, Altan M (2017). Effect of neoadjuvant chemotherapy on tumor-infiltrating lymphocytes and PD-L1 expression in breast cancer and its clinical significance. Breast Cancer Res.

[9] Mori H, Kubo M, Yamaguchi R (2017). The combination of PD-L1 expression and decreased tumor- infiltrating lymphocytes is associated with a poor prognosis in triple-negative breast cancer. Oncotarget.

[10] Galon J, Angell HK, Bedognetti D (2013). The continuum of cancer immunosurveillance: prognostic, predictive, and mechanistic signatures. Immunity.

[11] Salgado R, Denkert C, Demaria S (2015). The evaluation of tumor-infiltrating lymphocytes (TILs) in breast cancer: recommendations by an International TILs Working Group 2014. Ann Oncol.

[12] Chen DS, Mellman I (2017). Elements of cancer immunity and the cancer-immune set point. Nature.

[13] Liu F, Lang R, Zhao J (2011). CD8(+) cytotoxic T cell and FOXP3(+) regulatory T cell infiltration in relation to breast cancer survival and molecular subtypes. Breast Cancer Res Treat.

[14] Lazarevic V, Glimcher LH (2011). T-bet in disease. Nat Immunol.

[15] Lazarevic V, Glimcher LH, Lord GM (2013). T-bet: a bridge between innate and adaptive immunity. Nat Rev Immunol.

[16] Chen LJ, Zheng X, Shen YP (2013). Higher numbers of T-bet(+) intratumoral lymphoid cells correlate with better survival in gastric cancer. Cancer Immunol Immunother.

[17] Jrm Galon, Costes A, Sanchez-Cabo F (2006). Type, density, and location of immune cells within human colorectal tumors predict clinical outcome. Science.

[18] Origoni M, Parma M, Dell’Antonio G (2013). Prognostic significance of immunohistochemical phenotypes in patients treated for high-grade cervical intraepithelial neoplasia. Biomed Res Int.

[19] Mulligan AM, Raitman I, Feeley L (2013). Tumoral lymphocytic infiltration and expression of the chemokine CXCL10 in breast cancers from the Ontario Familial Breast Cancer Registry. Clin Cancer Res.

[20] Mulligan AM, Pinnaduwage D, Tchatchou S (2016). Validation of intratumoral T-bet^+^ lymphoid cells as predictors of disease-free survival in breast cancer. Cancer Immunol Res.

[21] NCCN Clinical Practice Guidelines in Oncology, Breast Cancer, Version 3 (2018). https://www.nccn.org/professionals/physician_gls/pdf/breast.pdf. Accessed 27 Dec 2018

[22] Goldhirsch A, Wood WC, Coates AS (2011). Strategies for subtypes–dealing with the diversity of breast cancer: highlights of the St. Gallen International Expert Consensus on the Primary Therapy of Early Breast Cancer 2011. Ann Oncol.

[23] Goldhirsch A, Ingle JN, Gelber RD (2009). Thresholds for therapies: highlights of the St Gallen International Expert Consensus on the primary therapy of early breast cancer 2009. Ann Oncol.

[24] Goldhirsch A, Wood WC, Gelber RD (2007). Progress and promise: highlights of the international expert consensus on the primary therapy of early breast cancer 2007. Ann Oncol.

[25] Goldhirsch A, Glick JH, Gelber RD (2005). Meeting highlights: international expert consensus on the primary therapy of early breast cancer 2005. Ann Oncol.

[26] Clinical Practice Guideline of Breast Cancer by the Japanese Breast Cancer Society, Version 3 (2015) http://jbcs.xsrv.jp/guidline/. Accessed 27 Dec 2018. **In Japanese**

[27] Wolff AC, Hammond ME, Hicks DG (2013). Recommendations for human epidermal growth factor receptor 2 testing in breast cancer: American Society of Clinical Oncology/College of American Pathologists clinical practice guideline update. J Clin Oncol.

[28] Wolff AC, Hammond ME, Schwartz JN (2007). American Society of Clinical Oncology/College of American Pathologists guideline recommendations for human epidermal growth factor receptor 2 testing in breast cancer. J Clin Oncol.

[29] Pruneri G, Vingiani A, Bagnardi V (2016). Clinical validity of tumor-infiltrating lymphocytes analysis in patients with triple-negative breast cancer. Ann Oncol.

[30] Mahmoud SM, Paish EC, Powe DG (2011). Tumor-infiltrating CD8+ lymphocytes predict clinical outcome in breast cancer. J Clin Oncol.

[31] Baker K, Lachapelle J, Zlobec I (2011). Prognostic significance of CD8+ T lymphocytes in breast cancer depends upon both oestrogen receptor status and histological grade. Histopathology.

[32] Glaire M, Domingo E, Nicholson G (2018). Tumour-infiltrating CD8+ lymphocytes as a prognostic marker in colorectal cancer: a retrospective, pooled analysis of the QUASAR2 and VICTOR trials. J Clin Oncol.

[33] Liu S, Lachapelle J, Leung S (2012). CD8+ lymphocyte infiltration is an independent favorable prognostic indicator in basal-like breast cancer. Breast Cancer Res.

[34] Ali HR, Provenzano E, Dawson SJ (2014). Association between CD8+ T-cell infiltration and breast cancer survival in 12 439 patients. Ann Oncol.

[35] Lee M, Tayyari F, Pinnaduwage D (2018). Tumoral BRD4 expression in lymph node-negative breast cancer: association with T-bet^+^ tumor-infiltrating lymphocytes and disease-free survival. BMC Cancer.

[36] Szabo SJ, Kim ST, Costa GL (2000). A novel transcription factor, T-bet, Directs Th1 lineage commitment. Cell.

[37] Zhang XR, Zhang LY, Devadas S (2003). Reciprocal expression of TRAIL and CD95L in Th1 and Th2 cells: role of apoptosis in T helper subset differentiation. Cell Death Differ.

[38] Roepman P, Jassem J, Smit EF (2009). An immune response enriched 72-gene prognostic profile for early-stage non-small-cell lung cancer. Clin Cancer Res.

[39] Goodman AM, Piccioni D, Kato S (2018). Prevalence of PDL1 amplification and preliminary response to immune checkpoint blockade in solid tumors. JAMA Oncol.

[40] Ladoire S, Arnould L, Mignot G (2011). T-bet expression in intratumoral lymphoid structures after neoadjuvant trastuzumab plus docetaxel for HER2-overexpressing breast carcinoma predicts survival. Br J Cancer.

