# Supplementary figures and images for: T-bet+ lymphocytes infiltration as an independent better prognostic indicator for triple-negative breast cancer
Source: Breast Cancer Res Treat. 2019 May 8;176(3):569–77. doi: 10.1007/s10549-019-05256-2 (PMC6586701; doi:10.1007/s10549-019-05256-2)

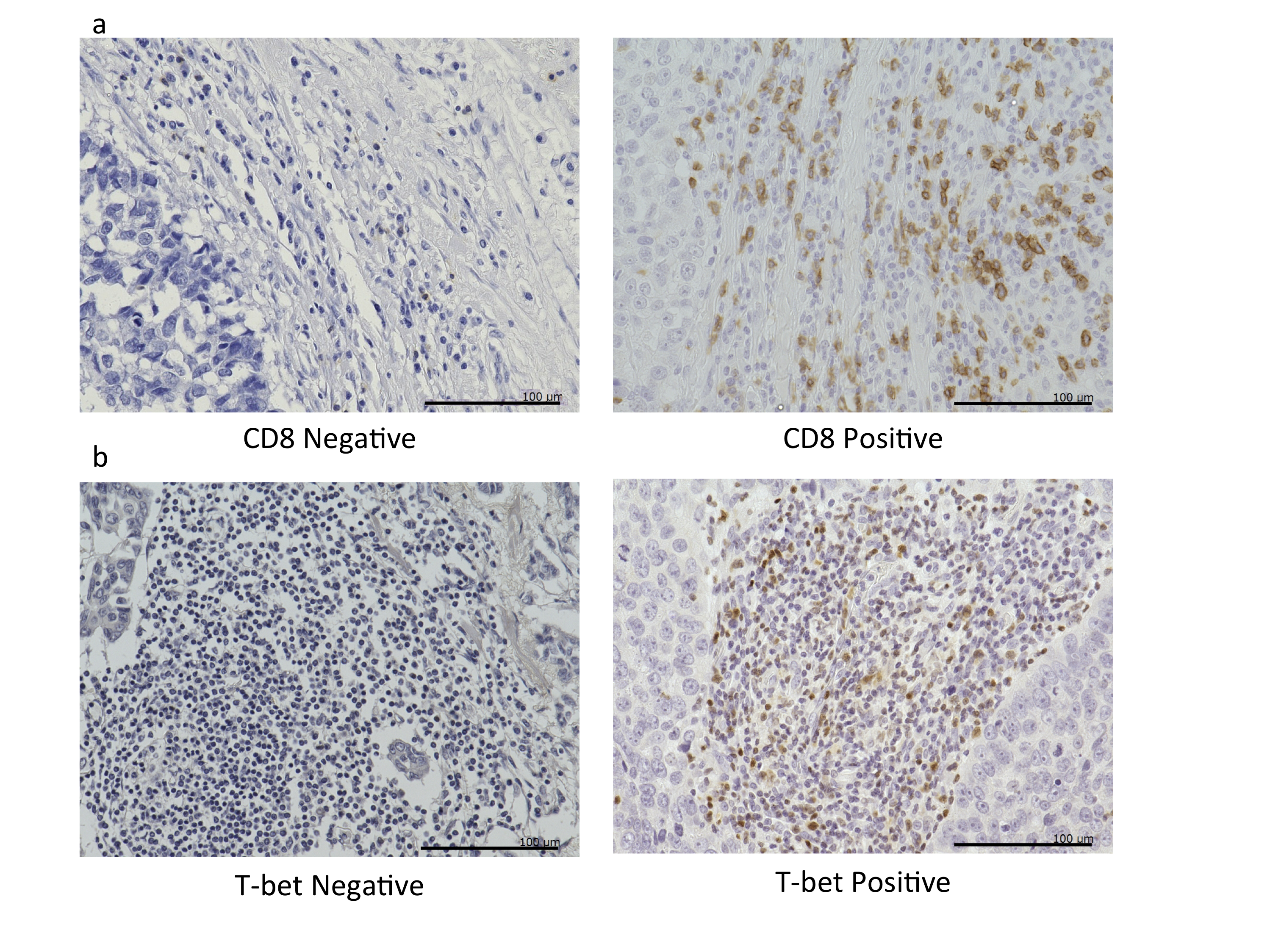

Supplement: Supplementary file 1 — Supplementary Fig. S1: Immunostaining of CD8 and T-bet on TILs (×400). (TIFF 26,330 kb) [file 10549_2019_5256_MOESM1_ESM.tiff]

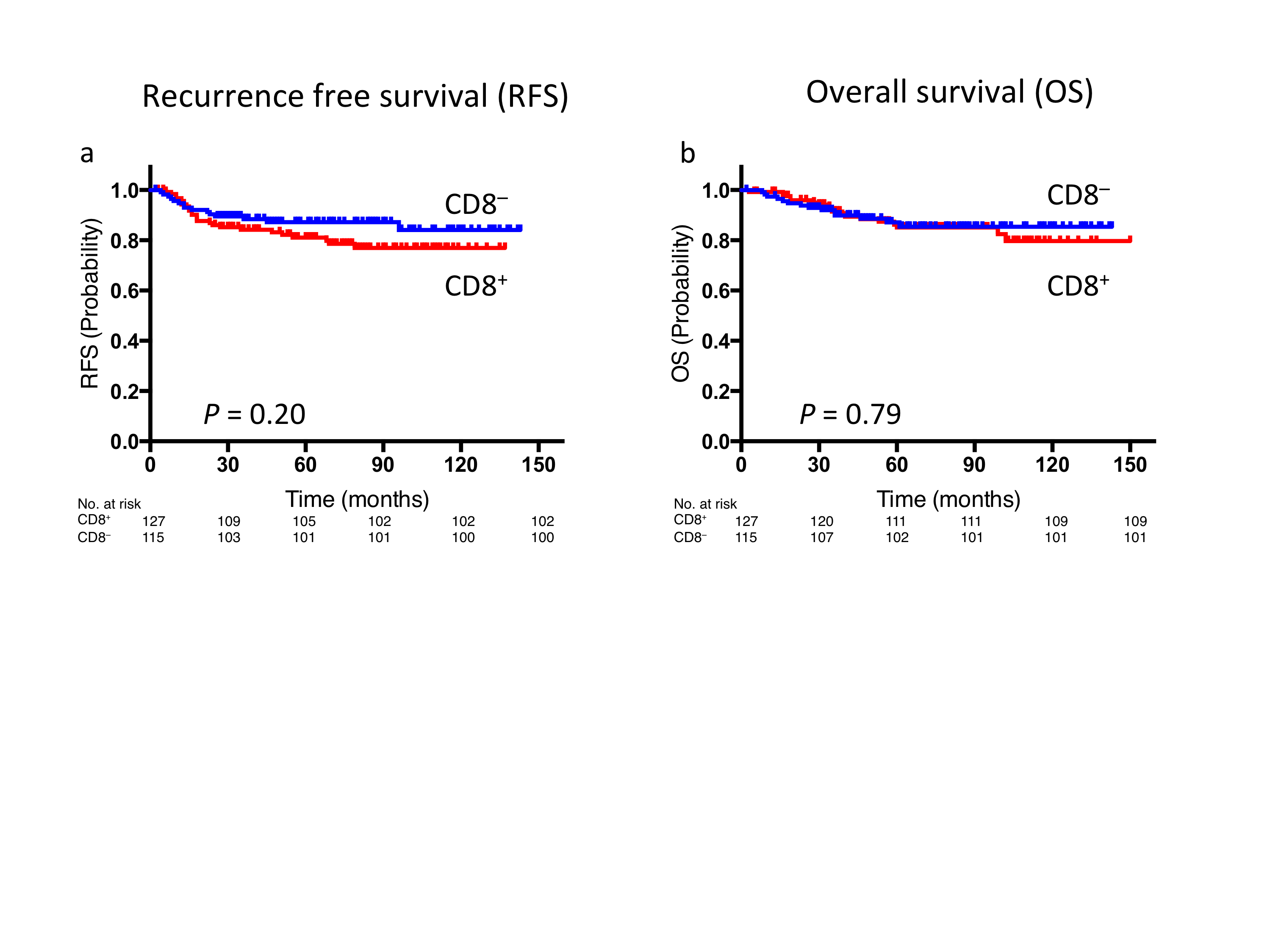

Supplement: Supplementary file 2 — Supplementary Fig. S2: Prognostic value of CD8 expression: Kaplan-Meier curves showing estimated RFS (a) and OS (b) for CD8 expression. p values are for comparison of two groups. (TIFF 26,330 kb) [file 10549_2019_5256_MOESM2_ESM.tiff]

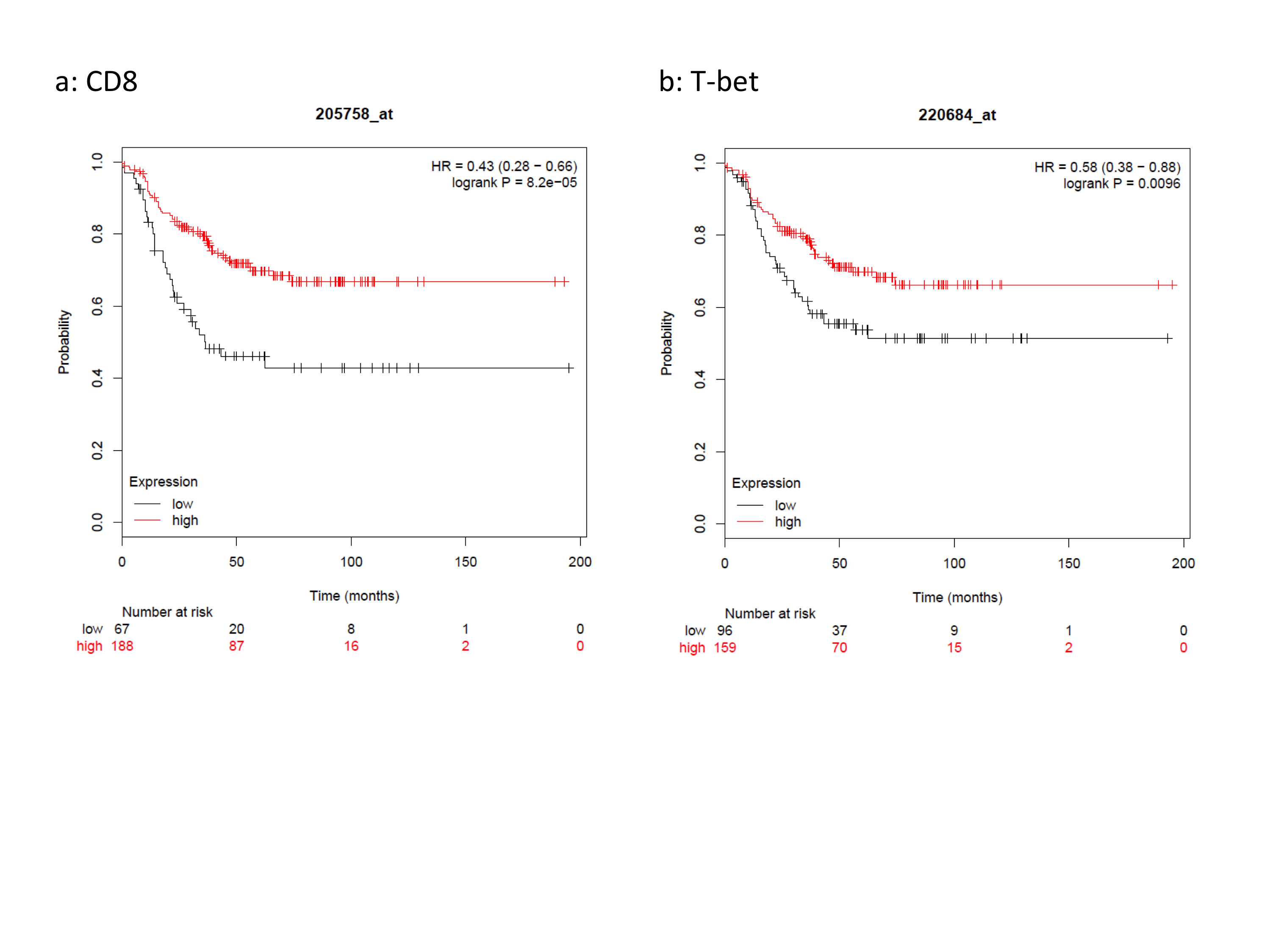

Supplement: Supplementary file 3 — Supplementary Fig. S3: Prognostic value of CD8 and T-bet mRNA expression in KM plotter (kmplot.com). Kaplan-Meier curve showing estimated RSF for CD8 (a) and T-bet (b) mRNA expression from triple negative breast cancer patients (N = 255) (TIFF 26,330 kb) [file 10549_2019_5256_MOESM3_ESM.tiff]
