# Supplementary material for: T-bet+ lymphocytes infiltration as an independent better prognostic indicator for triple-negative breast cancer
Source: Breast Cancer Res Treat. 2019 May 8;176(3):569–77. doi: 10.1007/s10549-019-05256-2 (PMC6586701; doi:10.1007/s10549-019-05256-2)
Supplement: Supplementary file 4 — Supplementary Table S1: Treatment characteristics for patients with TNBC. (DOCX 85 kb) [file 10549_2019_5256_MOESM4_ESM.docx]

| **Supplementary Table S1** Treatment characteristics for patients with TNBC | | | | | | | | | | |
| --- | --- | --- | --- | --- | --- | --- | --- | --- | --- | --- |
|  | CD8^+^ | | | |  | CD8^–^ | | | |  |
|  | T-bet^+^ | | T-bet^–^ | |  | T-bet^+^ | | T-bet^–^ | | *P* value |
| Surgical treatment |  |  |  |  |  |  |  |  |  |  |
| Breast-conserving surgery | 40 | (72.7%) | 38 | (52.8%) |  | 10 | (83.3%) | 56 | (54.4%) | **0.026** |
| Mastectomy | 15 | (27.3%) | 34 | (47.2%) |  | 2 | (16.7%) | 47 | (45.6%) |  |
| Adjuvant chemotherapy |  |  |  |  |  |  |  |  |  |  |
| Anthracycline-based regimens |  |  |  |  |  |  |  |  |  |  |
| AC, EC, FEC | 23 | (41.8%) | 22 | (30.6%) |  | 4 | (33.3%) | 36 | (35.0%) | 0.99 |
| EC+PTX, FEC+DTX | 15 | (27.3%) | 19 | (26.4%) |  | 5 | (41.7%) | 25 | (24.3%) |  |
| Non-anthracycline-based regimens | |  |  |  |  |  |  |  |  |  |
| TC, DTX | 1 | (1.8%) | 3 | (4.2%) |  | 0 |  | 4 | (3.9%) |  |
| CMF | 1 | (1.8%) | 2 | (2.8%) |  | 0 |  | 2 | (1.9%) |  |
| Others | 1 | (1.8%) | 2 | (2.8%) |  | 0 |  | 2 | (1.9%) |  |
| No treatment | 14 | (25.5%) | 23 | (31.9%) |  | 3 | (25.0%) | 34 | (33.0%) |  |
| Unknouwn | 0 |  | 1 | (1.4%) |  | 0 |  | 0 |  |  |

AC, doxorubicin (60mg/m^2^) and cyclophosphamide (600mg/m^2^) every 3 weeks; EC, epirubicin (90mg/m^2^) and cyclophosphamide (600mg/m^2^) every 3 weeks; FEC, 5-fluorouracil (500mg/m^2^), epirubicin (100mg/m^2^), and cyclophosphamide (500mg/m^2^) every 3 weeks; TC, docetaxel (75mg/m^2^) and cyclophosphamide (600mg/m^2^) every 3 weeks; DTX, docetaxel (75mg/m^2^) every 3 weeks; CMF, cyclophosphamide (100mg/m^2^), methotrexate (40mg/m^2^), and 5-fluorouracil (600mg/m^2^) every 4 weeks.
