# Supplementary material for: T-bet+ lymphocytes infiltration as an independent better prognostic indicator for triple-negative breast cancer
Source: Breast Cancer Res Treat. 2019 May 8;176(3):569–77. doi: 10.1007/s10549-019-05256-2 (PMC6586701; doi:10.1007/s10549-019-05256-2)
Supplement: Supplementary file 5 — Supplementary Table S2: Patient and tumor characteristics based on CD8 expression. (DOCX 104 kb) [file 10549_2019_5256_MOESM5_ESM.docx]

| **Supplementary Table S2** Patient and tumor characteristics based on CD8 expression. | | | | | | | |
| --- | --- | --- | --- | --- | --- | --- | --- |
|  | CD8^+^ | |  | CD8^–^ | |  |  |
|  | *N* = 127 (52.5%) | |  | *N* = 115 (47.5%) | | *P* value |  |
| Age at diagnosis |  |  |  |  |  |  |  |
| Mean (range) | 59.2 | (31-87) |  | 61.1 | (30-89) | 0.24 | ^a)^ |
| Tumor size | | | | | | | |
| T1a/b (≤ 1 cm) | 12 | (9.4%) |  | 8 | (7.0%) | 0.24 | ^b)^ |
| T1c (> 1 cm, ≤ 2 cm) | 69 | (54.3%) |  | 52 | (45.2%) |  |  |
| T2 (> 2 cm, ≤ 5 cm) | 44 | (34.7%) |  | 50 | (43.5%) |  |  |
| T3 (> 5 cm) | 2 | (1.6%) |  | 5 | (4.3%) |  |  |
| Nodal status | | | | | | | |
| N0 | 83 | (65.4%) |  | 79 | (68.7%) | 0.91 | ^b)^ |
| N1 (1−3) | 33 | (26.0%) |  | 25 | (21.7%) |  |  |
| N2 (4−9) | 7 | (5.5%) |  | 6 | (5.2%) |  |  |
| N3 (≥ 10) | 4 | (3.1%) |  | 4 | (3.5%) |  |  |
| Unknown |  |  |  | 0 | (0.9%) |  |  |
| Pathological stage | | | | | | | |
| I | 58 | (45.7%) |  | 44 | (38.3%) | 0.50 | ^b)^ |
| II | 59 | (46.5%) |  | 60 | (52.2%) |  |  |
| III | 10 | (7.9%) |  | 11 | (9.5%) |  |  |
| Nuclear grade | | | | | | | |
| 1+2 | 23 | (18.1%) |  | 47 | (40.9%) | **0.0001** | ^b)^ |
| 3 | 99 | (78.0%) |  | 66 | (57.4%) |  |  |
| Unknown | 5 | (3.9%) |  | 2 | (1.7%) |  |  |
| Ki-67 | | | | | | | |
| ≤ 30% | 16 | (12.6%) |  | 32 | (27.8%) | **0.022** | ^b)^ |
| > 30% | 84 | (66.1%) |  | 77 | (67.0%) |  |  |
| Unknown | 27 | (21.3%) |  | 6 | (5.2%) |  |  |
| T-bet | | | | | |  |  |
| Negative | 72 | (56.7%) |  | 103 | (89.6%) | **< 0.0001** | ^b)^ |
| Positive | 55 | (43.3%) |  | 12 | (10.4%) |  |  |
| Surgical treatment | | | | | |  |  |
| Breast-conserving surgery | 78 | (61.4%) |  | 66 | (57.4%) | 0.53 | ^b)^ |
| Mastectomy | 49 | (38.6%) |  | 49 | (42.6%) |  |  |
| Adjuvant chemotherapy | | | | | |  |  |
| Anthracycline-based regimens | | | | | | 0.97 | ^b)^ |
| AC, EC, FEC | 45 | (35.4%) |  | 40 | (34.8%) |  |  |
| EC+PTX, FEC+DTX | 34 | (26.8%) |  | 30 | (26.1%) |  |  |
| Non-anthracycline-based regimens | | | | | |  |  |
| TC, DTX | 4 | (3.1%) |  | 4 | (3.5%) |  |  |
| CMF | 3 | (2.4%) |  | 2 | (1.7%) |  |  |
| Others | 3 | (2.4%) |  | 2 | (1.7%) |  |  |
| No treatment | 37 | (29.1%) |  | 37 | (32.2%) |  |  |
| Unknouwn | 1 | (0.8%) |  | 0 |  |  |  |
| ^a)^ Logistic regression, ^b)^ Pearson's χ^2^ test. | | | | | | | |

AC, doxorubicin (60mg/m^2^) and cyclophosphamide (600mg/m^2^) every 3 weeks; EC, epirubicin (90mg/m^2^) and cyclophosphamide (600mg/m^2^) every 3 weeks; FEC, 5-fluorouracil (500mg/m^2^), epirubicin (100mg/m^2^), and cyclophosphamide (500mg/m^2^) every 3 weeks; TC, docetaxel (75mg/m^2^) and cyclophosphamide (600mg/m^2^) every 3 weeks; DTX, docetaxel (75mg/m^2^) every 3 weeks; CMF, cyclophosphamide (100mg/m^2^), methotrexate (40mg/m^2^), and 5-fluorouracil (600mg/m^2^) every 4 weeks.
