# Supplementary material for: T-bet+ lymphocytes infiltration as an independent better prognostic indicator for triple-negative breast cancer
Source: Breast Cancer Res Treat. 2019 May 8;176(3):569–77. doi: 10.1007/s10549-019-05256-2 (PMC6586701; doi:10.1007/s10549-019-05256-2)
Supplement: Supplementary file 6 — Supplementary Table S3: Interaction between T-bet and other factors in a Cox proportional hazards model (DOCX 66 kb) [file 10549_2019_5256_MOESM6_ESM.docx]

| **Supplementary Table S3: Interactions between T-bet and other factors in a Cox proportional hazards model** | | | | | | | | | | | | | | | | | | | | | | | |  |  | |  | | | |  |  |
| --- | --- | --- | --- | --- | --- | --- | --- | --- | --- | --- | --- | --- | --- | --- | --- | --- | --- | --- | --- | --- | --- | --- | --- | --- | --- | --- | --- | --- | --- | --- | --- | --- |
| **a**. T-bet and CD8 | | |  | | |  | | | | | |  | | | |  | |  | | | |  |  | |  |  | | |  |  | | |
|  | Recurrence-free survival | | | | | | | | | | | | | Overall Survival | | | | | | | | | | |  |  | |  |  |  |  |  |
|  | Estimated  value | | | Standard  error | | | 95% CI | | |  | Estimated  value | | | | | | Standard  error | | | 95% CI | | | | |  |  |  |  |  |  |  |  |
| T-bet (CD8 = Positive) | 0.53 | | | 0.24 | | | **0.08−1.03** | | |  | 0.67 | | | | | | 0.30 | | | **0.13−1.32** | | | | |  |  |  |  |  |  |  |  |
| T-bet (CD8 = Negative) | -0.04 | | | 0.29 | | | -0.60−0.55 | | |  | 0.23 | | | | | | 0.35 | | | -0.44−0.97 | | | | |  |  |  |  |  |  |  |  |
|  | | |  | | |  | | | | | |  | | | |  | |  | | | |  |  | |  |  | | |  |  | | |
| **b.** T-bet and Adjuvant treatment | | | | | | | | | | | | | | | | | | | | | | | | | | | | | | | |  |
|  | | | Recurrence-free survival | | | | | | Overall Survival | | | | | | | | | | | | | | | |  |  |  |  |  |  |  |  |
|  | | Estimated  value | | | Standard  error | | | 95% CI | | | | |  | | Estimated  value | | | | Standard  error | | 95% CI | | | |  |  |  |  |  |  |  |  |
| Adj. (T-bet = Positive) | | -0.73 | | | 0.32 | | | **-1.36− -0.10** | | | | |  | | -1.10 | | | | 0.34 | | **-1.77− -0.42** | | | |  |  |  |  |  |  |  |  |
| Adj. (T-bet = Negative) | | 0.11 | | | 0.20 | | | -0.26−0.51 | | | | |  | | -0.17 | | | | 0.21 | | -0.58−0.25 | | | |  |  |  |  |  |  |  |  |

Adj., Adjuvant treatment
